# Gut Microbiota in Acute Ischemic Stroke: From Pathophysiology to Therapeutic Implications

**DOI:** 10.3389/fneur.2020.00598

**Published:** 2020-06-25

**Authors:** Denise Battaglini, Pedro Moreno Pimentel-Coelho, Chiara Robba, Claudia C. dos Santos, Fernanda Ferreira Cruz, Paolo Pelosi, Patricia Rieken Macedo Rocco

**Affiliations:** ^1^Anesthesia and Intensive Care, San Martino Policlinico Hospital, IRCCS for Oncology and Neuroscience, Genoa, Italy; ^2^Department of Surgical Sciences and Integrated Diagnostics, University of Genoa, Genoa, Italy; ^3^Laboratório de Neurobiologia Comparada e do Desenvolvimento, Carlos Chagas Filho Institute of Biophysics, Federal University of Rio de Janeiro, Rio de Janeiro, Brazil; ^4^Keenan and Li Ka Shing Knowledge Institute, University Health Toronto—St. Michael's Hospital, Toronto, ON, Canada; ^5^Laboratory of Pulmonary Investigation, Carlos Chagas Filho Institute of Biophysics, Federal University of Rio de Janeiro, Rio de Janeiro, Brazil; ^6^Rio de Janeiro Network on Neuroinflammation, Carlos Chagas Filho Foundation for Supporting Research in the State of Rio de Janeiro (FAPERJ), Rio de Janeiro, Brazil

**Keywords:** microbiome, acute ischemic stroke, inflammation, dysbiosis, microbiota

## Abstract

The microbiota–gut–brain axis is considered a central regulator of the immune system after acute ischemic stroke (AIS), with a potential role in determining outcome. Several pathways are involved in the evolution of gut microbiota dysbiosis after AIS. *Brain–gut* and *gut–brain* signaling pathways involve bidirectional communication between the hypothalamic–pituitary–adrenal axis, the autonomic nervous system, the enteric nervous system, and the immune cells of the gut. Alterations in gut microbiome can be a risk factor and may also lead to AIS. Both risk factors for AIS and gut-microbiome composition are influenced by similar factors, including diabetes, hypertension, hyperlipidemia, obesity, and vascular dysfunction. Furthermore, the systemic inflammatory response after AIS may yield liver, renal, respiratory, gastrointestinal, and cardiovascular impairment, including the multiple organ dysfunction syndrome. This review focus on biochemical, immunological, and neuroanatomical modulation of gut microbiota and its possible systemic harmful effects after AIS, as well as the role of ischemic stroke on microbiota composition. Finally, we highlight the role of gut microbiota as a potential novel therapeutic target in acute ischemic stroke.

## Introduction

Acute ischemic stroke (AIS) is the second leading cause of death worldwide, accounting for up to 25% of global lifetime risk (1). Great effort has been invested into identifying risk factors, elucidating pathogenesis, and discovering implications for outcomes (2). Post-AIS infection has been identified as a key cause of death and prolonged hospitalization after stroke (3). Recent advances have demonstrated, for instance, that peripheral adaptive immunity is activated and recruited into the brain within the first few hours/days after AIS (4), and that its cells might regulate and be regulated by the gut microbiota (5). A microbiota is defined as an ecological unit composed of microorganisms within a specific (micro) environment, while the microbiome is the genetic material of these microorganisms (6). Dysbiosis is defined as a microbial imbalance in composition and function of the microbiota, occurring in several animal models of AIS which demonstrates that gut microbiota can regulate the neuroinflammatory response, influencing brain recovery (7). Several studies have focused on the relationship between the intestinal microbiome and AIS, confirming the existence of a bidirectional microbiota–gut–brain axis (8). In fact, alterations in gut microbiome can be a risk factor for AIS, and vice-versa; AIS may lead to changes in gut microbiome, impacting on peripheral organs and leading to severe liver, renal, respiratory, gastrointestinal and cardiovascular impairment, including the multiple organ dysfunction syndrome (MODS) (9). The aim of this review is to highlight the pathophysiology potentially involved in gut microbiota modulation after AIS, and its implication for therapy and outcome.

## Pathophysiology

### Brain–Gut–Microbiota Axis

#### Communication Pathways

**Figure 1:**
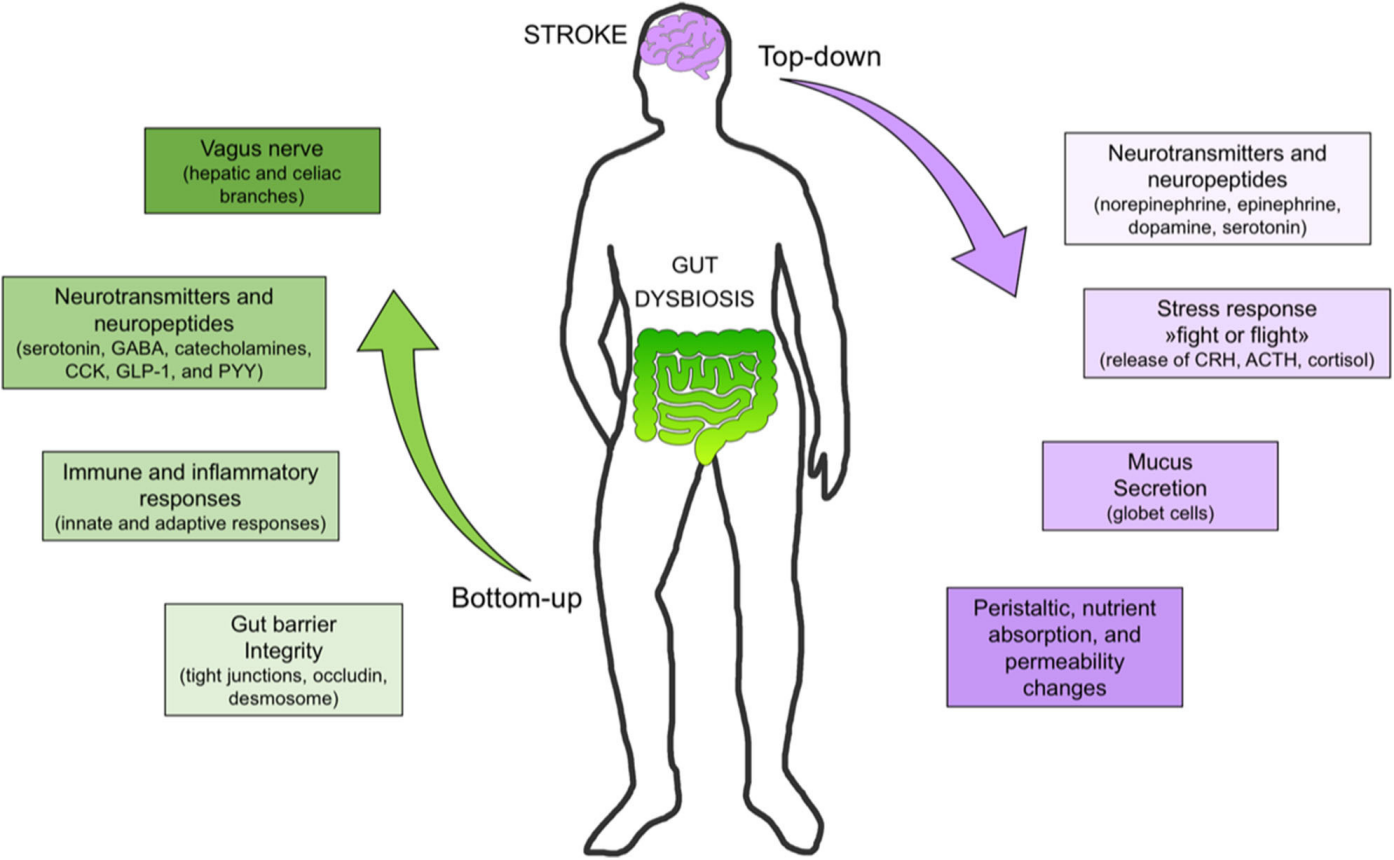
Bottom-up and top-down signaling in stroke. Bottom-up signaling from the gut to the brain includes barrier integrity maintenance, immune response (e.g., immunoglobulin A secretion), neurotransmitter and neuropeptide release, short-chain fatty acid (e.g., butyrate) release, and vagus nerve activation. Top-down signaling includes release of neurotransmitters (e.g., dopamine, serotonin), stress response (e.g., cortisol release), mucus secretion, and peristalsis control. [SCFA, short chain fatty acid; DA, dopamine; 5HT, serotonin; Ig, immunoglobulin, PYY, peptide YY; GLP-1, glucagon-like peptide-1; CKK, cholecystokinin; GABA, gamma amino butyric acid].

Recent evidence confirms the existence of bidirectional communication pathways linking the brain and the gut. The pathways involved in brain-gut axis include sympathetic and parasympathetic activation, the hypothalamic–pituitary–adrenal axis, and the immune system at a central level (8). The hypothalamic–pituitary–adrenal axis is a key communication mechanism between the brain and gut, particularly in response to a variety of stressful and stimuli (10). The autonomic nervous system, after integrating neuronal and neuroendocrine signaling, modulates intestinal homeostasis, thus enhancing inflammation in gut tissue, reducing the number of goblet cells in the cecum, and impairing mucin production in the overall intestine (8). The autonomic system is further responsible for controlling gut motility, permeability, fluid maintenance, bile secretion, bicarbonate and mucus production, intestinal fluid handling, and the mucosal neuroimmune response (11). Peripheral connections from the gut to the brain consist of the enteric and autonomic nervous systems, alongside the autonomic nervous system and various neuroimmune and neuroendocrine pathways (Figure 1) (8). These include serotonin, gamma-amino butyric acid (GABA), catecholamines (12), cholecystokinin, glucagon-like peptide-1, and neuropeptide Y (7). Bottom-up signals triggered upon stimulation of hepatic and celiac branches of the *vagus* nerve by microbial compounds, metabolites, and hormones released from the gut are carried by afferent vagal branches to the brain (13).

### Gut Dysbiosis and Neurological Disorders

Molecular biology has made great strides in characterizing which microorganisms co-inhabit each host and how this community may change over time. Each individual shows a unique microbiota profile with specific functions, such as immunomodulation, protection against pathogens, maintenance of gut mucosal barrier integrity, and control of nutrient metabolism (14). The largest population of commensal bacteria, comprising more than 1,000 species, is located in the digestive tract (15). Mucosal bacteria are more interactive with host cells when compared to luminal bacteria, influencing host gene expression and healing (16). This occurs, in part, due to the presence of the intestinal mucus layer, an essential component of the protective barrier between the intestinal epithelium and the gut lumen (17). The gut microbiota is composed of several bacterial species, of which 90% is represented by *Firmicutes* and *Bacteroidetes*. The *Firmicutes* phylum is predominantly composed of clostridial genera (14). Dysbiosis is usually caused by several mechanisms, such as infection, inflammation, diet, xenobiotics, and genetics, although the full list of lifestyle factors involved in microbiota modulation is still unknown (18). Inflammation compromises the microbiotic defense against pathogens causing dysbiosis, since the innate and adaptive immune systems are essential in regulation of microbiota homeostasis (19). The enteric nervous system is composed of the submucosal and the myenteric plexuses, which control intestinal motility and fluid movement (20). Their function is influenced by the activation of pattern recognition receptors (PRRs), especially toll-like receptors (TLRs) (21). Innate immunity primarily recognizes gut microbes by pathogen-associated molecular patterns (PAMPs), which are PRRs. The stimulation of PAMPs such as TLR-2 is responsible for hippocampal neurogenesis, while TLR-4 exhibits the opposite mechanisms (22). TLR-4 is involved in the development of learning and memory (23), and inhibits retinal neurogenesis and differentiation through pathways mediated by myeloid differentiation primary response protein (MYD)-88 and nuclear factor-κB (NF-κB) (24). The loss of MYD-88 in epithelial cells yields increased bacterial translocation and altered bacterial microbiota (25). TLR-5 controls intestinal microbial ecology, preventing dysbiosis (26). Nucleotide-binding oligomerization domain-containing protein (NOD)-like receptors (NLRs) are also determinants of microbial dysbiosis. Among NOD receptor subtypes, NOD1 is implicated in binding peptidoglycan from Gram-negative bacteria (27). NOD2 is involved in increased burden of commensals and mucosa-associated bacteria (28), its absence predisposing to dysbiosis. In contrast, NOD6 is involved in the maintenance of a stable intestinal microbial community (29). NOD2, which is responsible for intestinal homeostasis, activates kinase receptor-interacting protein (RIPK)-2 and NF-kB, which are involved in the control of the production of antimicrobial peptides (AMPs) and mucin. AMP expression is also influenced by flagellin and lipoproteins, which interact with TLR-5 on dendritic and epithelial cells (29). Additionally, some NLR proteins create a multiprotein complex known as the inflammasome. The inflammasome activates caspase-1, which processes interleukin (IL)-1β and IL-18 precursors. Overall, inflammasome activation results in IL-1β and IL-18 secretion and changes in several immune cell populations (30). Regarding the adaptive immune response, compelling evidence suggests that B cells play an important role in microbiota homeostasis through the secretion of immunoglobulin (Ig)A (31). IgA selection is promoted by T helper follicular cells. This mechanism of IgA secretion is regulated by death protein-1, which manages the microbiome at the intestinal level (32). Invariant natural killer T-cells are also involved in microbiota regulation (33). Specifically, a symbiotic relationship between the immune system in the intestine and the microbiota exists. In fact, the intestinal immune system protects the organism against pathogens germs, while the microbiota maintains the intestinal immune system, inducing secretion of IgG from plasma cells, IL-17- and IL-22-from helper T cells (Th-17 cells), which are situated in the mucosal lamina propria, and represent the mediators of microbiota-immune system crosstalk. IgA directly promotes colonization by mutualistic microbes, competing with invasive pathogens. IL-17 and IL-22 maintain the mucosal barrier system homeostasis, which protects the organism stimulating the expression anti-microbial molecules in intestinal epithelial cells. Moreover, T regulator (Treg) cells contribute to immune homeostasis in non-lymphoid tissues including the gastrointestinal tract, recruited by T-cell antigen receptor (TCR) and cytokine signals expression, differentiating into suppressive effector Treg cells with IL-10 production. IL-10 holds anti-inflammatory effect, contributing to preserve gut homeostasis. Dysbiosis is correlated to an increased susceptibility to intestinal inflammation, because of reduced IgA, IL-22, and IL-10 production, and IL-17R impairment (34). The activation of innate and adaptive immune responses at mucosal surfaces during inflammation, autoimmunity, and infection explains why microbiota composition could play an essential role in modulating immune response in other organs, such as the brain (35). Evidence from germ-free mice suggests correlations among an immature microglial phenotype, altered immune response, and brain pathology. This can be modulated by short-chain fatty acids and microbiota-derived bacterial fermentation products (36). Microglia are essential for the maintenance of tissue homeostasis, synaptic remodeling, and scavenging of pathogens, molecules and death cells, and have been associated with neuropsychiatric and neurological disease in humans (36). Under germ-free conditions, microglial polarization (especially M1>M2) is reduced, suggesting that reduced complexity of microbiota impairs microglial function (36). Several neurological diseases correlate with blood–brain barrier impairment, in which microbiota dysregulation may play a role. These include Parkinson's disease, Alzheimer's disease, several mental disorders, and autism spectrum disorders (24). Experimental research has hypothesized that increased blood–brain barrier permeability might be induced by the loss of normal microbiota diversity (37). Besides, microbiota diversity is not only associated with blood–brain barrier permeability, but also plays a crucial role in hippocampal and microglial brain morphology (24). Additionally, several neurological disorders are associated with an enhanced local inflammatory response, which can contribute to systemic cytokine release and increased tissue permeability, thus altering microbiota homeostasis (24). In fact, dysbiosis of the intestinal microbiota in neurocritically ill patients has been correlated to mortality at 180 days (38).

### The Brain–Gut–Microbiota Axis in AIS

Local and systemic inflammatory responses are enhanced after AIS (4). Monocytes (innate response) enter the brain within 24 h, reaching maximum numbers around 3–5 days after AIS (39). They differentiate into macrophages with a distinct molecular signature compared to microglia (40). Inflammatory monocytes have been shown to exert protective effects following AIS in mice (41), whereas the role of patrolling monocytes is less clear (42). Neutrophils play a controversial role in AIS. Although several mechanisms (43) (such as the production of reactive oxygen species and the release of metalloproteinases) have been attributed to neutrophils, the existence of neuroprotective N2 neutrophils has also been reported (44). A recent study in mice identified the triggering receptor expressed on myeloid cells (TREM)-1 as an interesting target for pharmacotherapies aimed at reducing the pro-inflammatory response of peripheral and intestinal myeloid cells after AIS (45).

**Figure 2:**
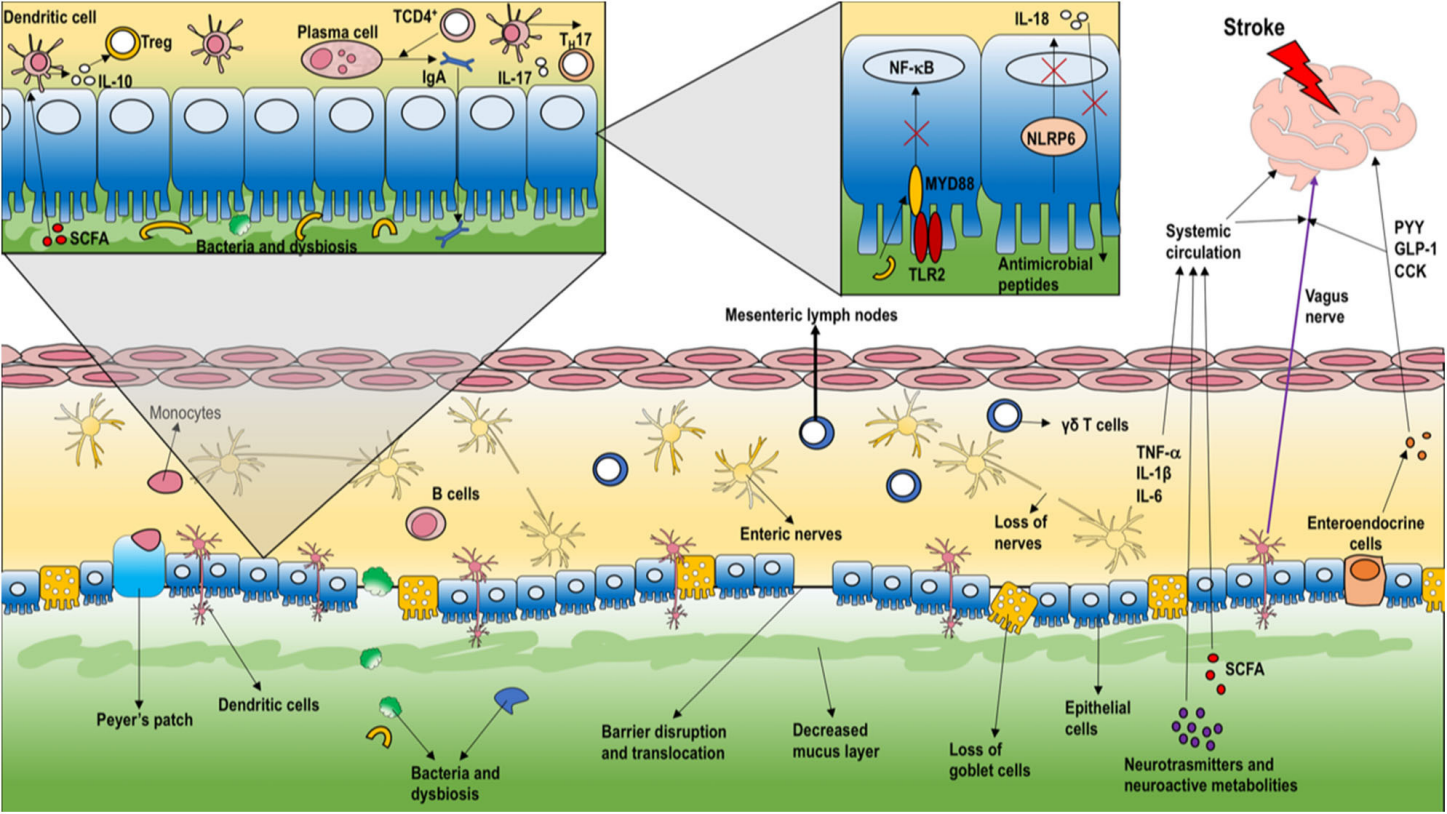
Local intestinal inflammatory response in stroke. As the first intestinal response to AIS, the enteric barrier is disrupted, thus compromising the ability of the microbiota to defend against intestinal pathogens. The microbiota stimulates release of metabolites, neurotransmitters, indoles, short-chain fatty acids, and bile acids that can reach the brain, thus modulating neurons, microglia, astrocytes, and neurovascular unit function. Vagal afference activate neuroendocrine system to release peptides [peptide YY (PYY), glucagon-like peptide-1 (GLP-1), cholecystokinin (CCK)], as well as inflammatory response to enhance pro-inflammatory cytokines release (e.g., interleukin (IL)-21) from cluster of differentiation (CD)-4^+^ T-cells and IL-17 from γδT-cells. IL-17 acts to recall neutrophils by releasing chemokines. Moreover, T lymphocytes can migrate from the Peyer patches of the small intestine or from the intestinal lamina propria to the brain and/or the leptomeninges. As depicted in the inset, the loss of myeloid differentiation primary response protein (MYD)-88 in epithelial cells results in increased bacterial translocation and altered bacterial microbiota. Moreover, nucleotide-binding oligomerization domain-containing protein (NOD)-like receptors-6 (NLRP6) are involved in the maintenance of a stable intestinal microbial community. Usually, NLR proteins create a multiprotein complex named the inflammasome, activating IL-1β and IL-18 secretion and changes in several immune cell populations. B-cells also play an important role in microbiotic homeostasis through secretion of immunoglobulin A, promoted by TCD4+ on plasma cells. Additionally, dendritic cells are stimulated by T regulatory cells to increase the secretion of IL-10. [PYY, peptide YY; GLP-1, glucagon-like peptide-1; CCK, cholecystokinin; IL, interleukin; CD, cluster of differentiation; MYD, myeloid differentiation primary response protein; NOD, nucleotide binding oligomerization domain-containing protein; NLRP, nucleotide-binding oligomerization domain-containing protein receptor; CRH, corticotropin releasing hormone; ACTH, adrenocorticotropic hormone].

TREM1 is a potent enhancer of innate immune responses, it acts by synergizing with classical PRRs, thus inducing the production of proinflammatory cytokines and chemokines, including IL-8, monocyte chemoattractant protein-1 (MCP-1), MCP-3 and macrophage inflammatory (MIP-1α), and inhibition of IL-10 (45). Furthermore, neurotoxic mechanisms activate the release of pro-inflammatory cytokines such as interleukin-21 from cluster of differentiation (CD)-4^+^ T cells within 24 h after AIS (46) and IL-17 from γδT-cells (47). IL-17 acts by recalling neutrophils through chemokine release, whereas IL-21 and perforin exert direct neurotoxic effects in the brain (48). Clinical trials have failed to provide evidence of beneficial effects of neutrophil blockade in AIS patients (49). γδT-cells express the chemokine receptor (CCR)-6, which is essential for their infiltration into the AIS lesion (47). In the first 2-3 days after AIS, lymphocytes arrive at the ischemic lesion (adaptive response) (4). In particular, T helper-1 and-17 subpopulations activate neuroinflammation, whereas regulatory T-cells have a neuroprotective action due to their anti-inflammatory properties (50). The consequences of this remain unclear, as detrimental effects to regulatory T cells after AIS have been reported (51). Release into the systemic circulation of cytokines and chemokines produced in the brain after AIS may exert a potential influence on microbiota composition and dysbiosis (52). T lymphocytes, especially regulatory and γδT-cells, play a pivotal role in how the microbiota can modify infarct size and neurological function after AIS (7, 53). T lymphocytes have been shown to migrate from the Peyer patches of the small intestine or from the intestinal lamina propria to the brain and/or the leptomeninges following AIS (7). Increased gut permeability after AIS can result in bacterial translocation and lung infection in mice (17). Local inflammatory responses at the intestinal level in AIS are depicted in Figure 2. Despite some degree of controversy arising from study heterogeneity, pneumonia—presumably secondary to immune dysregulation and bacterial translocation—is the most common acute complication after AIS (54). Immunogenic endotoxins from the microbiota, such as lipopolysaccharide (LPS), can promote neuroinflammation by a direct mechanism and/or by inducing migration of peripheral immune cells to the brain (55). An interesting experimental study showed that AIS in cynomolgus monkeys induced a long-term, persistent increase in levels of LPS and pro-inflammatory cytokines in plasma, which correlated with the relative abundance of the phylum Bacteroidetes in the gut microbiota. Additionally, intestinal dysbiosis and mucosal damage persisted for up to 12 months after AIS (52). Clinical evidences (56, 57) concluded that after AIS the commensal flora changes in favor of opportunistic pathogens, such as *Enterobacter, Megasphaera, Oscillibacter, Desulfovibrio, Odoribacter*, and *Akkermansia* (58), instead of commensals such as *Bacteroides, Prevotella*, and *Faecalibacterium*. Although several mechanisms involved in the microbiota-regulated immune response after AIS have been identified, our understanding about microbiota modulation following AIS is incompletely understood.

## Clinical Implications

**Figure 3:**
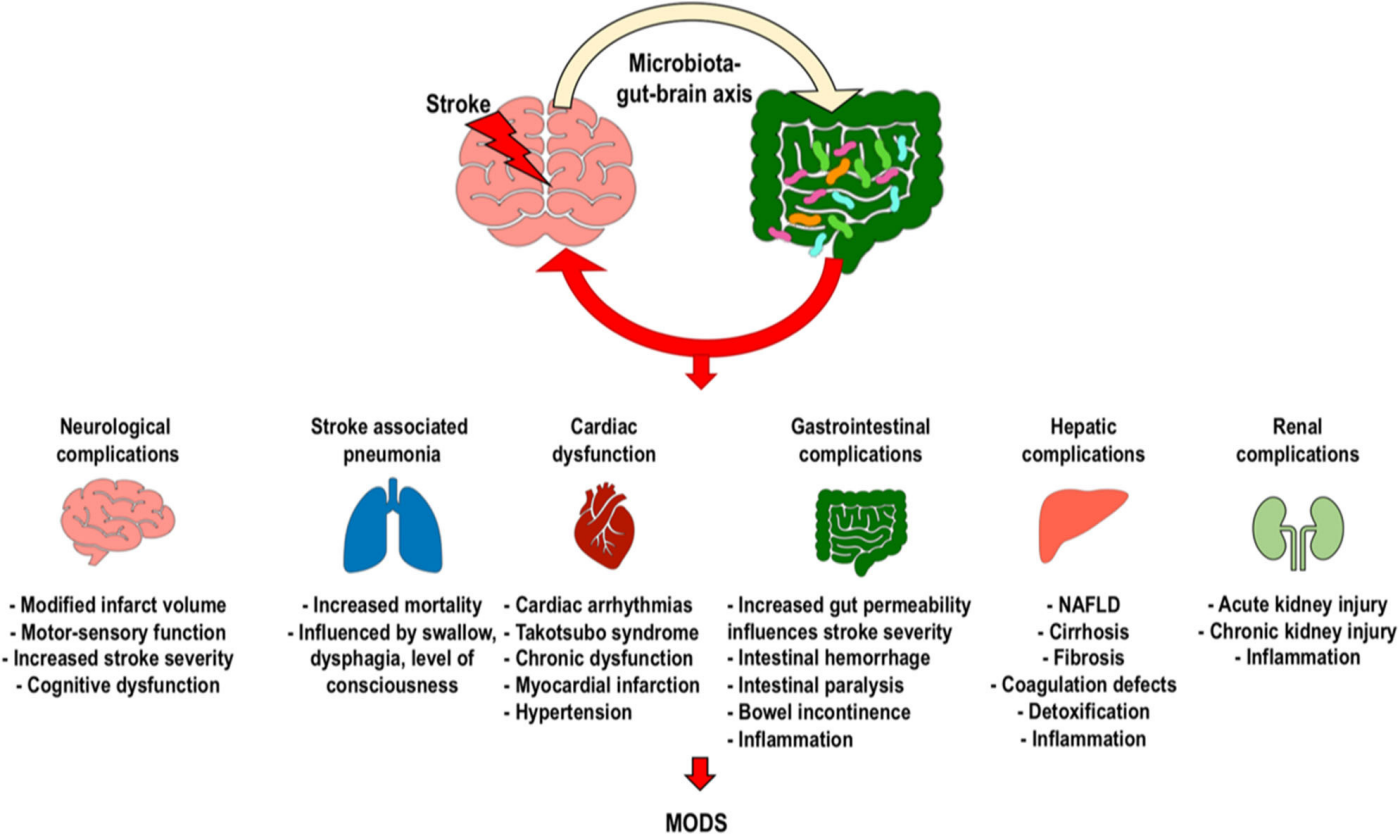
Clinical implications of intestinal dysbiosis after ischemic stroke. Changes caused by stroke on the gut microbiota can induce neurological complications, stroke-associated pneumonia, cardiac dysfunction, gastrointestinal complications, and renal dysfunction, with possible development of the systemic inflammatory response and multiple organ dysfunction syndromes. [MODS, multiple organ dysfunction syndrome].

Alterations in the gut microbiota composition have been identified in various neurological diseases, including cognitive dysfunction, autism, neurodegenerative disorders, and cerebrovascular diseases (24). The gut microbiome is pivotal for brain function and behavior, as demonstrated by the fact that gut dysbiosis is associated with impairment of the blood–brain barrier, behavioral deficits, and alterations of synaptic plasticity (59). We will discuss how alterations in the gut microbiota during AIS affect neurologic outcomes, risk factors, and occurrence of AIS-associated pneumonia, as well as cardiovascular, gastrointestinal, hepatic, renal complications and development of the multiple organ dysfunction syndrome, thus potentially affecting clinical outcomes and functional recovery (Figure 3) (6).

### Influence of Gut Dysbiosis on AIS Outcomes

Gut dysbiosis in AIS patients is associated with host metabolism and inflammation (60, 61). Increased levels of opportunistic pathogens in the gut have been associated with higher risk of developing AIS (62). A clinical study that compared asymptomatic controls to patients with AIS reported significant changes in the gut microbiota composition in terms of increased opportunistic pathogens, with this finding persisting for at least 3 weeks after AIS (57). Gut dysbiosis was measured in AIS patients by developing a score called the “stroke dysbiosis index (SDI),” which has shown good correlation in predicting severe stroke and unfavorable outcome (56).

### AIS Risk Factors and Gut Dysbiosis

AIS patients often show significant changes in microbial flora that may be independent of relevant comorbidities (hypertension, age, and type 2 diabetes) (56). However, alterations in the gut microbiota are known to elicit hypertension in rats, raising doubts as to the preceding evidence (63). Like hypertension, obesity and body composition in both sexes are associated with metabolic syndrome and higher cardiovascular and cerebrovascular risk, which are influenced by inflammation and gut permeability, thereby suggesting a role of gut microbiota in the prevention of these diseases (64).

#### Hypertension

Diabetes and hypertension have been identified as systemic activators of immune response and are two of the most important risk factors for AIS (65). Intensive management of hypertension in AIS patients is recommended to reduce the risk of chronic myocardial dysfunction and cardiac remodeling (66–68). Hypertensive and pre-hypertensive patients have a different gut microbiota composition as compared to non-hypertensive patients. The finding of altered microbiota composition in pre-hypertensive patients suggests that dysbiosis most commonly appears before clinical hypertension, rather than hypertension being the causative factor of dysbiosis (63).

#### Obesity and Diabetes Mellitus

These metabolic disorders are known to increase the risk of cerebrovascular diseases, such as AIS (6). The gut microbiota plays paramount roles in the regulation of satiety, thermogenesis, and energy balance, due to its ability to manage host immune cell activation and maturation and neuro-endocrine and hormonal functions. Furthermore, dysbiosis activates pro-inflammatory mechanisms that are implicated in diabetes and cardiovascular disease. In particular, trimethylamine N-oxide (TMAO) may be implicated in the pathogenesis of cardiovascular, metabolic and cerebrovascular diseases (69). In fact, in patients with type I diabetes, higher concentrations of plasma TMAO were associated with mortality, cardiovascular events, and micro and macrovascular complications (70). Elevated TMAO levels within the first hours after AIS are associated with poor stroke outcomes (71).

#### Vascular Dysfunction

Vascular dysfunction can influence mortality and outcome following AIS. Bacterial metabolites such as short-chain fatty acids, nitrites, flavanol, TMAO, indoles, and sulfidic acid have been identified as causative factors of vascular dysfunction. Short chain fatty acids and sulfidic acid are particularly capable of inducing vasodilatation, whereas indole and trimethylamine N-oxide increase the production of reactive oxygen species, thus reducing cerebral vasodilatation (63, 72).

#### Aging

According to recent investigations, the gut microbiota undergoes several modifications during the life course. Specifically, immune function, microbiota variability, and anti-inflammatory properties are reduced in older adults.

### AIS-Associated Pneumonia

The mechanism underlying immune dysregulation and reduced antimicrobial defense after AIS is still unclear. Infection rates range between 5 and 65%; this variability is attributed to the heterogeneity of the current definition (73). Stanley et al. suggested a role for bacterial translocation in the pathogenesis of AIS-associated pneumonia (17). Pneumonia is a typical example of dysbiosis-related infection. Pneumonia correlates with an increased mortality rate (73). A recent meta-analysis including 137,817 AIS patients from 87 studies confirmed an overall infection incidence of 30%, with pneumonia accounting for up to 10% (74). Dysphagia occurs in 50–55% of AIS patients (75), and the incidence of AIS-associated aspiration pneumonia ranges from 4 to 57% (76). *Staphylococcus aureus, Klebsiella pneumoniae, Pseudomonas aeruginosa, Escherichia coli*, and *Enterobacter* species are commonly identified, especially in the intensive care unit (77). Another important factor leading to pneumonia after AIS is immunosuppression (4). In post-AIS mice, aspiration of only 200 colony-forming units of *Streptococcus pneumoniae* was enough to trigger pneumonia and bacteremia compared to sham-operated mice, in which up to 20,0000 colony forming units were required to induce pneumonia (78).

### Cardiovascular Complications

The incidence of cardiovascular events is increased after AIS (79), accounting for up to 39% of complications in AIS patients without previous cardiac events (80). The most common disturbances include cardiac arrhythmias, stress-induced cardiomyopathy (Takotsubo syndrome), autonomic dysfunction, myocardial infarction, and arterial hypertension (81). According to recent evidence, the occurrence of cardiovascular and cerebrovascular diseases correlates with dysbiosis (82). TMAO, a byproduct of the intestinal microbiota, is a potential novel biomarker of cardiovascular events, including AIS (83), and also associated with poor outcomes (84).

### Gastrointestinal Complications

Water and nutrients are absorbed by the gut–blood barrier, which also prevents the passage of toxins and pathogens into the blood (85). Intestinal permeability is modified by AIS-induced inflammation, thus enhancing microbiota dysregulation (85). Moreover, increased intestinal permeability has been associated with greater AIS severity (85), as well as imbalance of the intestinal flora in favor of pathogens, thereby influencing outcome and post-AIS mortality (86). After AIS, up to 50% of patients develop gastrointestinal complications such as hemorrhage, intestinal paralysis, bowel incontinence, and dysphagia, which are considered partially responsible for poor neurological outcomes and increased mortality (53, 87). This deterioration of outcomes is mainly attributed to increased immune activation in the gut, with systemic migration of lymphocytes to the brain (53). It is interesting to note that patients with Crohn's disease have an increased susceptibility to AIS, further implying a bidirectional association between gut dysbiosis and AIS (88).

### Hepatic Complications

The potential association between liver disease, AIS, and gut microbiota dysregulation is poorly understood (89). Since the systemic coagulation cascade is managed mostly by pro- and anticoagulant factors produced by the liver, hepatic dysfunction can cause coagulopathies with bleeding or thrombotic complications, and has consequently been identified as an important risk factor for AIS (90). Among hepatic disorders, non-alcoholic fatty acid disease (NAFLD), cirrhosis, and liver fibrosis are the most common factors associated with AIS (89, 91). The liver plays a major role in the biotransformation of drugs and toxins. Trimethylamine is converted by hepatic enzymes into TMAO, which is also implicated in platelet hyperreactivity, atherosclerosis (92), inflammation, and cholesterol metabolism (93). The gut microbiota is able to modulate cholesterol transport by activating of specific enzymes that suggests a role in modulation of risk factors involved in the development of AIS (94).

### Renal Complications

Risk factors for kidney injury and AIS are very similar, and atherosclerosis plays a crucial role in both contexts (95). In a recent meta-analysis, the incidence of acute kidney injury in AIS patients reached up to 9.6%, and was associated with increased overall mortality rate (96), in-hospital mortality, and neurological deterioration (97). Uremic toxins are waste products of microbiota that can cross the intestinal blood–barrier because of increased gut permeability after AIS, and thus reach the systemic circulation (98). In support of a purported gut–brain–kidney axis, an experimental study demonstrated that rats affected by both acute kidney injury and AIS displayed neuronal loss; glial, macrophage, and microglial upsurge; and increased circulating IL-6 and IL-1β levels (99). Following a systemic inflammatory process, high level of microbiota waste products such as TMAO are found in patients with chronic kidney disease, suggesting that TMAO is inversely associated with glomerular filtration rate and should be considered as a new clinical marker of renal medullary damage, hypertension, and heart disease (100). TMAO plasma concentrations are elevated in patients with chronic kidney disease, and predict poor long-term survival; in animal models, TMAO is associated with tubulointerstitial fibrosis and renal dysfunction (101).

### Multiple Organ Dysfunction Syndrome

The gut is the most colonized system of the body. It is home to a broad range of commensal and pathogenic microorganisms—up to trillions of different types of bacteria. The intestinal mucosal barrier activates a specialized local immune response due to the existence of organized gut-associated lymphoid tissue (GALT) (102). Leaky gut (i.e., increased intestinal permeability) can determine the release of intestinal lumen bacteria and toxins into the circulation, thus enhancing systemic inflammation and causing sepsis or a systemic inflammatory response (9). Indeed, the systemic inflammatory response syndrome has been detected as a complication of systemic inflammation after AIS (9). A study conducted on 1,500 AIS patients revealed that systemic inflammation at admission was associated with infarct volume, functional outcome, and clinical severity (103). Moreover, within the first days after AIS, white blood cell counts, body temperature, and C-reactive protein levels were higher; therapeutic thrombolysis attenuated this inflammatory response (103). Systemic inflammatory response can result in MODS in up to 12% of AIS patients, increasing the mortality rate up to 80%. (104) Among risk factors for MODS after AIS, a low Glasgow Coma Score, advanced age, hypoglycemia, hyperglycemia, leukocytosis, and history of chronic organ dysfunction have been identified (104). Unfortunately, clinical data on multiorgan failure and systemic inflammatory response in AIS patients is scant, and their correlation with microbiota manipulation poor.

## Therapeutic Strategies

### Diet

Diet is a major determinant of gut microbiota composition. Indeed, dietary diversity has been associated with regulation of insulin resistance, susceptibility to infections, and TMAO concentration, among other changes (105). The production of TMAO by gut microbiota can be increased by high intake of L-carnitine and phosphatidylcholine, which is an excellent dietary source of choline commonly found in red meat and eggs (106). Moreover, antibiotic treatment has been found to reduce TMAO generation, whereas, discontinuation of antibiotics caused an increase in TMAO levels (84). High-fat diets such as the ketogenic diet [commonly used for its antiseizure properties by increasing serum ketones and reducing neuronal apoptosis (107)] have also been found to increase TMAO concentration in humans (108), whereas the Mediterranean diet and vegetarian diets reduced TMAO production (94). The Mediterranean diet has been widely investigated in recent decades for its potential health benefits. Indeed, the consumption of cereals, nuts, vegetables, legumes, fruit, and fish can mitigate the incidence of neurodegenerative disorders, psychiatric diseases (109), and cardiovascular dysfunction (110). A study reported that patients with higher Mediterranean Diet Score Stratification had a lower *Firmicutes/Bacteroidetes* ratio, thus suggesting anti-inflammatory effects of the Mediterranean diet and its modulation of gut microbiota (111). Protein-rich diets have been associated with conflicting results. Although amino acids are considered essential for synthesis of neurotransmitters (112), long-term adherence to a high-protein diet is associated with harmful effects on gut microbiota composition (113). There has been growing interest in the role of dietary fiber in gut microbiota composition. Fiber is mainly fermented by *Firmicutes* and other bacterial species, thus increasing the production of short chain fatty acids (SCFAs) such as acetate, propionate, and butyrate (114). SCFAs have also been suggested to improve post-stroke recovery via immune mechanisms (115). There is little evidence on the impact of fiber-rich diets on AIS outcome, but, as demonstrated in experimental settings, the immunomodulatory effect of fibers seems to be anti-inflammatory.

### Probiotics and Prebiotics

Probiotics are defined as “live micro-organisms which induce benefits in the host,” and although they are largely assumed to be safe and useful, four broad categories of adverse effects have been identified: (1) systemic infections, (2) deleterious metabolic activities, (3) excessive immune stimulation, and (4) undesired gene transfer (116). Importantly, in immunocompromised patients, probiotic administration has been associated with the development of sepsis (116). On the other hand, probiotics act through acid lactic fermentation in the gut, thus improving the balance between pathogens and commensals, enhancing immune regulation of the intestinal system, and inhibiting bacterial toxin production (117). Prebiotics are not digested in the small bowel, but are active in the colon, where they are fermented by bacteria. Carbohydrates are an example of prebiotics (117). Prebiotics are capable of influencing the production of SCFAs and regulating mucin production and local inflammatory response into the GALT, thus stimulating phagocytosis by macrophages (117). In a meta-analysis of thirteen clinical trials, treatment with prebiotics reduced neither intensive care unit mortality nor in-hospital mortality. However, administration of prebiotics resulted in declines in both incidence of pneumonia associated with intensive care unit stay and in-hospital length-of-stay (118). Therefore, based on this limited clinical evidence, prebiotics might be hypothesized to reduce the risk of pneumonia, which is particularly high in AIS patients (73).

### Antibiotics

To date, no clear evidence is available in support of prophylactic antibiotic treatment within the first hours after AIS to control dysbiosis (77). A recent 1,224-patient study demonstrated that prophylactic antibiotics administered immediately following AIS, and primarily in those patients affected by post-AIS dysphagia, did not reduce the incidence of pneumonia (119). A second prospective randomized study confirmed a reduction in overall infections after prophylactic antibiotic therapy with 2 g of intravenous ceftriaxone every 24 h for 4 days relative to standard care, but this did not affect the incidence of post-AIS pneumonia or functional outcome scores at 2 months (120). In 2017, a randomized controlled trial of 227 patients compared standard care plus ultrasensitive procalcitonin-guided antibiotic treatment to standard care alone. When procalcitonin reached a value greater than 0.05 ng/ml, prophylactic antibiotics were administered following local guidelines. There were no beneficial effects on functional outcome at 90 days, nor on mortality rate (77).

### Fecal Transplantation, Intra-Gastric Treatments, and Microbial Metabolites

#### Fecal Transplantation

Gut microbiota dysbiosis can influence the severity of brain injury (7). The effect of intestinal bacteria on neuronal function is defined as *psychobiotics*. Fecal microbiota transplantation is the transfer of donor fecal microbiota from healthy people to sick patients (121), and it has recently been identified as a possible strategy to correct dysbiosis in patients with neuropsychiatric disorders and those affected by AIS (122). Antibiotic administration reduced neurological impairment, blood lipid levels, and infarct volume, while transplanting stool rich in SCFAs (especially butyric acid) led to microbiota remodeling, increasing *Lactobacillus* species and improving intestinal microbiota, thus positively modulating the brain ischemic response (123). Fecal microbiota transplantation has proven effective for intestinal dysbiosis in clinical settings, although its use was associated with secondary bacteremia from *E. coli* infection in two patients with recurrent *Clostridioides difficile* infection, transmitted by the donor fecal microbiota (124). Nevertheless, this treatment is recognized as a novel alternative to antibiotic therapies against primary *C. difficile* infection in small cohorts of patients (124). Taken together, these studies suggest that fecal transplantation can broadly affect intestinal microbiota in recipients.

#### Intragastric Treatments and Microbial Metabolites

Intragastric treatment with *Clostridium butyricum* after bilateral common carotid artery occlusion resulted in reduced neuronal damage and cognitive impairment in rodents (125). These beneficial effects might be at least partially related to production of the microbial metabolite butyrate, which has been shown to be neuroprotective in models of AIS (126). In another study in rats, sodium butyrate mitigated blood–brain barrier permeability following AIS by reducing the activity of matrix metalloproteinase-9 (127). Among metabolites, GABA plays an important role in tryptophan–tryptamine–serotonin metabolism. Tryptophan is a precursor of both host and microbial metabolites (such as kynurenic acid (128) and quinolinic acid, which is associated with neurodegenerative diseases by neurotoxic modulation) with anti-inflammatory and neuroprotective properties (129). Further studies are underway and future research will be needed to reveal the impact of fecal microbiota manipulation and metabolite activity on neuronal function.

### Environment

The environment has a great impact on daily life and health. The “halogenome theory of evolution” suggests that both the host species and the symbiotic microbiota, which together form a unit known as the *halobiont*, exert genetic selection in response to environmental demand (e.g., stress, temperature, diet, industry, etc.) (6). An example of harmful environment is contact with heavy metals like cadmium, mercury, and arsenic, which can modify the immune system towards nuclear changes and disturb gut microbiota composition (6). Likewise, bisphenol A, a compound widely used by the plastic industry, can disrupt intestinal microbiota with adverse effects (6). Antibiotic abuse can increase their levels in the environment, contaminating the soil, bodies of water, and waste, thus causing huge consequences at the global level (6).

**Figure 4:**
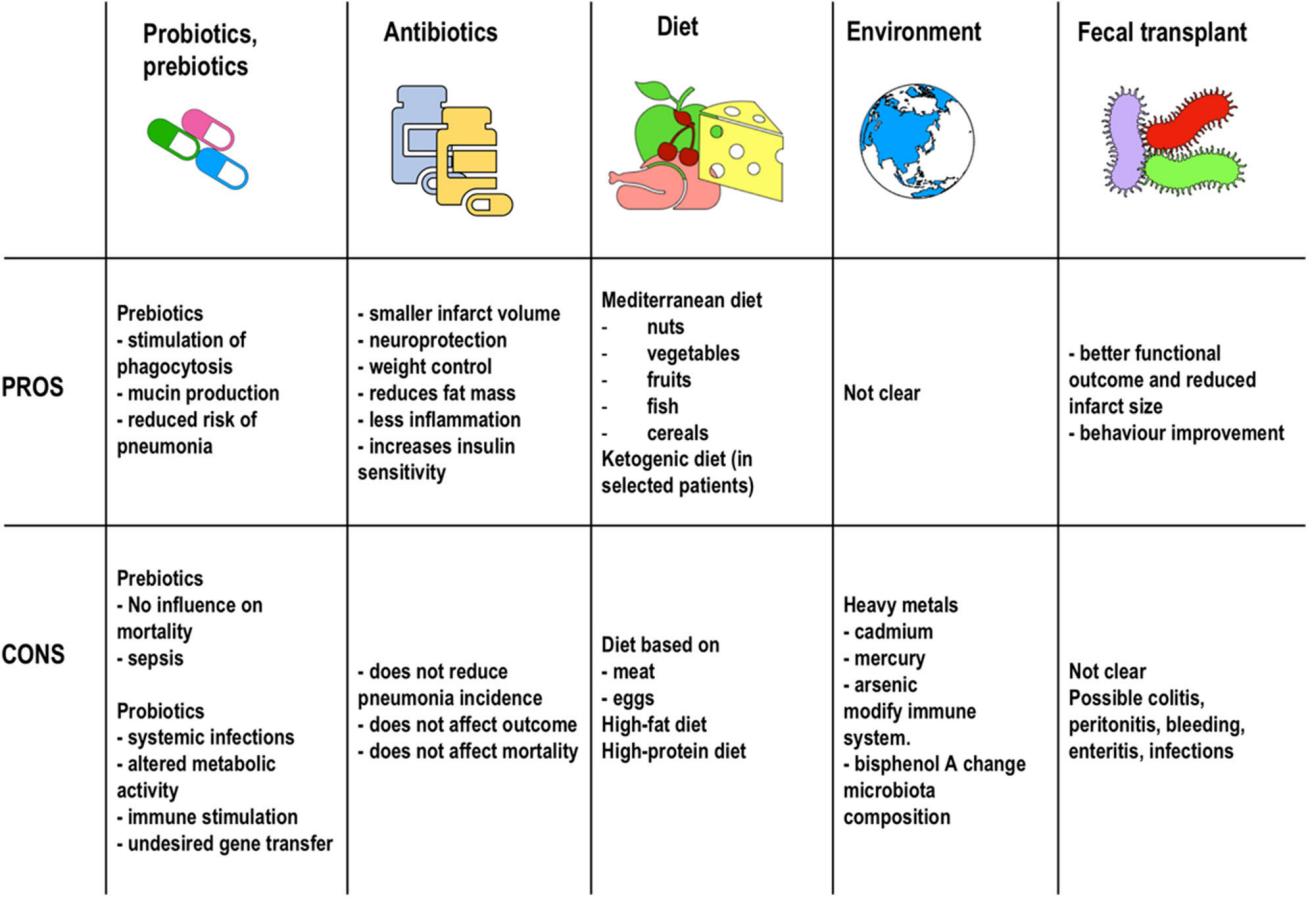
Therapeutic strategies. Pros and cons of the therapeutic strategies described in the literature to date for microbiota manipulation in stroke patients.

In summary, various factors such as diet, probiotics, prebiotics, antibiotics, and the environment seem to be associated with meaningful changes in the gut microbiota. Alterations in both function and composition of this microbiota seems to profoundly affect risks and outcomes in AIS patients. As we move forward, the challenge will be to determine causal relationships and develop strategies to optimize microbiota composition in order to reduce risk and modulate outcomes and recovery (Figure 4).

## Conclusions

Translational microbiome research against the enhanced systemic inflammatory immune and neuroendocrine responses and on the impact of modulation of the environment, diet, and drugs on the so-called halobiont in AIS patients are limited. Since only few of these studies have demonstrated that antibiotic treatment, probiotics, exercise, or environmental changes could be essential for microbiota and outcome modulation, microbiota dysregulation after AIS remains a challenging target for new therapies.

## Author Contributions

DB wrote the manuscript. DB, PR, PP-C, and FC designed the review. PP-C, FC, CS, PP, and PR revised the manuscript. All authors contributed to the article and approved the submitted version.

## Conflict of Interest

The authors declare that the research was conducted in the absence of any commercial or financial relationships that could be construed as a potential conflict of interest.

## Abbreviations

AMPs: antimicrobial peptides
AIS: acute ischemic stroke
CCR: chemokine receptor
CD: cluster of differentiation
GABA: gamma-aminobutyric acid
GALT: gut-associated lymphoid tissue
Ig: immunoglobulin
IL: interleukins
LPS: lipopolysaccharide
MCP: monocyte chemoattractant protein
MODS: multiple organ dysfunction syndrome
MYD: myeloid differentiation primary response protein
NAFLD: non-alcoholic fatty acid disease
NF-kB: nuclear factor-kappa B
NLRs: nucleotide-binding oligomerization domain-containing protein receptors
NOD: nucleotide-binding oligomerization domain-containing protein
PAMPs: pathogen associated molecular pattern
PRRs: pattern recognition receptor
RIPK: receptor-interacting protein kinase
SDI: stroke dysbiosis index
SCFA: short-chain fatty acid
TCR: T cell antigen receptor
TLRs: toll-like receptors
TMAO: trimethylamine N-oxide
Treg: T regulator lymphocytes
TREM: triggering receptor expressed on myeloid cells.
